# Introduction of the Thin Prep Imaging System™ (TIS): experience in a high volume academic practice

**DOI:** 10.1186/1742-6413-4-6

**Published:** 2007-02-08

**Authors:** Mamatha Chivukula, Reda S Saad, Esther Elishaev, Susan White, Nancy Mauser, David J Dabbs

**Affiliations:** 1 Department of Pathology, Magee-Womens Hospital of University of Pittsburgh Medical Center, Pittsburgh, PA, USA

## Abstract

**Objective:**

Since the introduction of the liquid-based ThinPrep testing in 1996, most cytology laboratories across the country have adopted the liquid-based cytology (LBC) for Pap test screening. Subsequent to wide-spread adoption of the ThinPrep Pap test, the ThinPrep Imaging System (TIS) Cytyc Corp, Marlborough, MA was introduced to improve the accuracy and efficiency of screening interpretation. We report our initial experience with the TIS at Magee Women's Hospital. We introduced the TIS in December 2004.

**Methods:**

The imager assisted Pap test results over the first 12 months (December 2004 to December 2005) of implementation were reviewed and analyzed. Our implementation protocol included each cytotechnologist manually prescreening 200 negative slides to gain experience with the imager slides and serve as a quality check for the TIS. We re-screened **3400 **slides (200 slides each for 17 cytotechnologists) manually which were initially determined to be negative using the TIS. **104,457 **Pap tests were imaged on the TIS. **95,899 **manually screened Pap tests, 12 months prior to the introduction of the TIS (December 2003–November 2004) are taken as the historic control group for our study.

**Results:**

The mean ASC-US rate employing the automated imager was **8.70% [9088/104,457]. **The mean LSIL detection rate was **4.22% [4409/104,457]. **The imager did not miss any detectible high-grade lesions during these months, with a HSIL (+) detection rate of **0.68% **in comparison to **0.60% **by manual screening confirmed by follow-up biopsies. The difference is statistically significant with a *p value *of *0.022*. The definition of false negative rate for purposes of this study is calculated as the number of false negative cases identified out of number of negatives re-screened. The TIS false negative rate was estimated at **0.012% [4/3400]**.

**Conclusion:**

The overall performance of the TIS in our lab appears to be highly satisfactory in terms of improving sensitivity in screening cervical precursor lesions. The increased accuracy of detection of HSIL indicates a positive impact of the TIS in our laboratory.

## Background

Primary cervical cancer screening techniques have undergone revolutionary changes since the introduction of conventional Pap smears in 1946. Liquid based Pap tests intended as a replacement for the conventional smears received FDA approval in 1996 for cervical cancer screening. In countries where cervical cancer screening is a routine procedure, manual screening of slides has always been a laborious task. There have been methods put forward to improve the assisted screening techniques. The Cytyc Corporation ThinPrep^® ^Imaging system (TIS), a computer imaging based technology, is one of the recent devices introduced to assist in primary cervical screening of ThinPrep Test slides for the presence of epithelial cell abnormalities as defined by the 2001 Bethesda System for Reporting Cervical Cytology (TBS)[1,2].

The TIS consists of an image processor connected to one or more review scopes. The review scope is a microscope connected to the imager with an automated stage and a control pad connected via Ethernet cables to enable locating 22 fields of interest. These fields are screened by the cytotechnologist for the presence of abnormal cells. The cytotechnologist can verify negative reports in the absence of abnormal cells, or rescreen the entire slide if there are any abnormal questionable cells detected by the TIS [2]. Of the centers which have validated and reported their experiences with the TIS imager, most are still in the form of abstracts [8-19], with available peer-reviewed papers reflecting data from the clinical trial studies [3,4]. A multi-center clinical trial has shown that the TIS is more sensitive than manual screening for Atypical Squamous Cells of Undetermined Significance or Higher [ASC-US (+)], while its sensitivity in the clinical trials was equivalent for Low grade squamous intraepithelial lesion (LSIL (+)) and High-grade squamous intraepithelial lesion (HSIL (+)) results. On the other hand, the specificity at the level of HSIL (+) exceeded that for ASC-US (+) and LSIL (+) [3]. The TIS was introduced in our lab recently in December 2004. Our study is one of the first to evaluate the initial performance of the TIS imager in a high volume academic hospital laboratory. Pap Tests that were manually screened during the 12-month period prior to the introduction of the TIS in our laboratory were used as the historical control group for our study.

## Materials and methods

The TIS was introduced in our hospital pathology laboratory in 2004. **104,457 **Pap tests were imaged by the TIS in the 12-month period, December 2004–December 2005. **95,899 **manually screened Pap tests, 12 months prior to the introduction of the TIS (December 2003–November 2004) are taken as the historic control group for our study. The salient features of both the comparison and the control groups include: 1) The TIS cohort and the control cohort are derived from the same practice and belong to the same demographic population. 2) The same cytotechnologists screened the Pap tests of both groups.

The cytological specimens collected routinely from the clinics were prepared using the ThinPrep 3000 Processor (Cytyc Corp) according to the manufacturer's instructions. Essentially, the vials containing the cells are placed in the processor and a dispersion cycle homogenizes the cell suspension. The cells are automatically collected on a polycarbonate filter membrane. A thin, evenly dispersed monolayer of cells is deposited from the filter onto the slide in a 20 mm circle. Extraneous mucus and blood are removed in the process. The slides are manually removed from the processor and stained by the Pap method. The specimen adequacy was recorded according to 2001 TBS guidelines [1]. When the TIS was introduced, our protocol initially included each cytotechnologist manually screening 200 slides to get acquainted with the imager slides. This also served as a quality check for the TIS imager. The definition of false negative rate for purposes of this study is calculated as the number of false negative cases identified out of number of negatives re-screened.

## Results

Among the **104,457 **Pap tests imaged, **1176 **were reported as unsatisfactory following the TBS criteria [1]. The mean rate of detection of ASC-US by the TIS was **8.70%. **The mean detection rate of LSIL by the TIS was **4.22% **and the ASCUS/LSIL ratio was **2.06%. **In the comparative group of the manual screening (MS) from the prior 12 months (January 04–December 04), **95,899 **Pap tests were manually screened of which 1725 were interpreted as unsatisfactory. The mean rate of detection of ASC-US by manual screening was **8.79%. **The detection rate of LSIL by manual screening was **3.90% **and the ASCUS/LSIL ratio was **2.25%. **There is an difference in the detection rates of LSIL by TIS. On the other hand there is a slight decrease in the detection rates of ASC-US and AGC categories There is an increase in the detection rates of ASC-H by the TIS as compared to MS.(0.6% vs 0.5%). The imager did not miss any detectible high-grade lesions, with detection rate of HSIL (+) of **0.68% **in comparison to **0.60 % **by manual screening. The difference is statistically significant with a *p value *of *0.022*. A comparison of the various abnormal interpretations by the TIS versus manual screening is shown in [Table 1, Fig 1]. The HSIL(+) Pap tests are confirmed by follow-up colposcopic biopsies. There is an overall increased rate of detection of cervical intraepithelial neoplasia (CIN) observed in the follow-up biopsies by **2%. **The increase is seen more so in the CIN 1 category [Table 3]. Follow-up biopsies results of ASC-H and HSIL (+) are shown in Tables 3, 4. A comparison of these tables show that there is certainly improved detection of CIN 1 w/HSIL + and ASC-H interpretation (**195 vs. 134 & 232 vs. 161**). There is also improved detection of CIN 2(+) by TIS (**281 vs 232**). In the follow-up biopsies of ASC-H cases, there is slightly decreased number of CIN 2(+) (**39 vs 45**). The two possible explanations for this might be a shift in the clinicians ordering HPV testing for ASC-H interpretation, that we are noticing now or these are more specifically interpreted as HSIL(+) (that can explain the increase in HSIL(+)rates). Overall there is an increase in ASC-H interpretation but at the expense of PPV for CIN 2(+) [Table 3].

**Table 1:** A comparison of various cytological diagnoses by Manual screening *vs*. TIS

	**Manual Screening [%]**	**TIS Screening [%]**
**ASC-US**	8.79 [8425/95,899]	8.70 [9088/104,457]
**LSIL**	3.90 [3744/95,899]	4.22 [4409/104,457]
HSIL (+)*	0.60 [577/95,899]	0.68 [714/104,457]
**ASC-H**	0.50 [475/95,899]	0.60 [634/104457]
**AGC**	0.30 [284/95,899]	0.28 [292/104,457]

* -HSIL (+) encompasses HSIL (CIN2.3), Carcinoma-in-situ (CIS) and invasive carcinoma (squamous & adenocarcinoma)

**Table 2:** Follow-up biopsy results of the missed cases by TIS

**Case #**	**Pap Test interpretation**	**F/u biopsy result**
**1**	Low-grade squamous intraepithelial lesion (LSIL)	Cervical intraepithelial neoplasia 1(CIN 1)
**2**	Low-grade squamous intraepithelial lesion (LSIL)	No follow-up biopsy
**3**	Low-grade squamous intraepithelial lesion (LSIL)	*No follow-up biopsy
**4**	Low-grade squamous intraepithelial lesion (LSIL)	ECC – Negative

*: Follow-up pap tests were ASCUS/HPV+ & LSIL (CIN 1)

**Table 3:** Biopsy follow-up of ASC-H category

**Year**	**Total# Bxs done**	**NEG**	**CIN 1**	**CIN 2+**	**No f/u**
**2005 (N = 634)**	414	143 **[34.5%]**	232 **[56%]**	39 **[9.4%]**	220 **[35%]**
**2004 (N = 475)**	232	133 **[57.3%]**	161 **[69.3%]**	45 **[19.4%]**	136 **[28.6%]**

**Table 4:** Biopsy follow-up of HSIL (+) category

**Year**	**Total# Bxs done**	**NEG**	**CIN 1**	**CIN 2+**	**No f/u**
**2005 (N-714)**	520	44 **[8.5%]**	195 **[38%]**	281 **[54%]**	194 **[27%]**
**2004 (N-577)**	408	42 **[10.3%]**	134 **[33%]**	232 **[57%]**	169 **[29%]**

Four cases were missed by the TIS with a false negative rate of **0.012 % (4/3400). **Of the four cases missed, two cases were rescreened by the cytotechnologist due to the previous history of abnormal pap. Two cases were detected by random screening by the cytotechnologist. The follow-up cervical biopsy in one case showed CIN1. Two cases did not have follow-up biopsies. In one case, an endocervical curettage (ECC) was performed and is negative [Table 2].

## Discussion

The methods of cervical cancer screening in the past 50 years have undergone major changes. The introduction of liquid based cytology screening and FDA approval of the HPV-DNA testing with the Pap test have added new dimension to the Pap test screening. The introduction of the ThinPrep method for LBC has shown superiority over conventional screening methods in several meta-analyses [5,7]. Reference studies have shown that there is more uniform cellular distribution, better morphological preservation and a cleaner background observed in the ThinPrep slides during the screening process [5].

The concept of computer assisted primary screening was to assist both screening accuracy and productivity [3]. The intent of the TIS was to assist the cytotechnologists in the difficult and tedious primary screening process [3]. Studies have been conducted to compare the image-assisted primary screening versus manual ThinPrep screening [8,9,13,14]. A multi-center two-armed clinical trial to evaluate the performance of the TIS revealed some promising results. The imager was found to have a significantly higher sensitivity in detection of ASC-US (+) with an increase of 6.4% as compared to the manual review method [3]. In our lab, the mean ASC-US rate and the ASCUS/LSIL rate showed a slight increase in comparison to the manual screening.

The detection rates of LSIL (+) in some studies on the TIS, showed a statistically significant increase in comparison to manual screening [14]. In our lab the mean LSIL rate (**4.08%) **by the TIS showed an increase by **8.2% **as compared to the control MS group **(4.03%)**. Our results are comparable with the other studies [3,8,12-14,18]. The most important clinical aspect of cervical cancer screening is detection of HSIL (+). In all the studies done so far on the TIS, there has been a noticeable increase in detection of HSIL (+) [3,9,13-15]. We now present a similar experience with the TIS in our lab. The detection rate of HSIL (+) by the TIS was **0.68% **which is more than by the MS **0.60%. **There is a significant increase in the detection rate noted by **13.3%. **The combined detection rates of Atypical squamous cells, cannot exclude HSIL (ASC-H) and HSIL (+) have also shown a significant increase by **16% **as compared to manual screening. We are reporting our initial experience with the TIS within one year of introduction using a ''*historical control*". We intend to report the trends in near future with more years of experience with TIS which would show more clearly the advantage of TIS.

With the implementation of the new imager, one of the changes included an adjustment by both the cytotechnologists and pathologists to the new stain. We have started using the new stain six months prior to the introduction of the TIS. Another important aspect of consideration was the workforce issues and the various turnaround time (TAT) parameters in the lab. One study has shown that the TIS has made a significant positive impact on TAT and workload [8]. In the near future we plan to look into the effect of introduction of the TIS on various TAT parameters.

The overall performance of the TIS in various clinical studies has demonstrated promising results. There appears to be an increased detection of ASC-US (+) and HSIL (+). In our laboratory the early evaluation of our experience with the TIS has been highly satisfactory. The TIS appears to be a promising tool in the cytology lab to improve the detection of cervical precursor lesions.

## Abbreviations used

MS- Manual screening; TAT- turn around time; LBC- Liquid-based cytology, CIN-Cervical intraepithelial neoplasia, TIS -Thin-Prep Imaging System

## Competing interests

The author(s) declare that they have no cometing interests.

## Authors' contributions

(MC): Faculty collected all the data, participated in cytological evaluation, and drafting of manuscript, presentation of the data at ASC conference.

(RS): Faculty, drafting of manuscript, manuscript writing.

(NM): Cytology Manager, Collection of data, drafting of manuscript, manuscript writing.

(EE): Faculty, drafting of manuscript, manuscript writing.

(SW): Cytotechnologist, Collection of data, drafting of manuscript, manuscript writing.

(DJD) Mentor, conceptual organization, cytological-histological evaluation, and manuscript writing.
